# Histopathological Alterations in the Livers of Chronic Hepatitis Patients Exposed to Agent Orange/Dioxin in Vietnam

**DOI:** 10.3390/toxics10060315

**Published:** 2022-06-10

**Authors:** Phu Quang Pham, Vuong Ba Nguyen, Tai The Pham, Nhuong Xuan Duong, Ha Trong Nguyen, Quang Van Ha, Thuan Duc Nguyen, Tuan Minh Hoang, Dong Tien Dinh, Quynh Thi Nhu Tran, Linh Kim Bui, Thuy Thi Vu, Manh Van Phan, Tuan Minh Luong, Khanh Nguyen, Dung Anh Vu, Thao Ngoc Pham

**Affiliations:** 1 Department of Gastroenterology, 103 Military Hospital, Vietnam Military Medical University, Hanoi 12108, Vietnam; bsphu79@yahoo.com (P.Q.P.); nhuongdx171@yahoo.com.vn (N.X.D.); drhoangtuanhvqy@gmail.com (T.M.H.); bsdonga1@gmail.com (D.T.D.); 2 Department of Haematology, Toxicology, Radiation and Occupation, 103 Military Hospital, Vietnam Military Medical University, Hanoi 12108, Vietnam; bshaa7103@gmail.com (H.T.N.); haquangss@gmail.com (Q.V.H.); nducthuanbv103@gmail.com (T.D.N.); 3 Biomedical and Pharmaceutical Research Centre, Vietnamese Military Medical University, Hanoi 12108, Vietnam; taithuy@kanazawa-med.ac.jp; 4 General Internal Medicine Department, National Hospital of Endocrinology, Hanoi 11500, Vietnam; tranquynh1785@gmail.com; 5 Department of Military Epidemiology, Vietnam Military Medical University, Hanoi 12108, Vietnam; builinhhvqy@gmail.com; 6 Department of Gastroenterology, Huu Nghi Hospital, Hanoi 11600, Vietnam; thuyvubvhuunghi@gmail.com; 7 Department of Military Hygiene, Vietnam Military Medical University, Hanoi 12108, Vietnam; bsphanvanmanh@gmail.com; 8 Army Medical Department, Ministry of Defence, Hanoi 11100, Vietnam; luongtuan27@gmail.com; 9 Department of Thoracic Surgeon, 103 Military Hospital, Vietnam Military Medical University, Hanoi 12108, Vietnam; drkhanh90@gmail.com; 10 Department of Orthopedic Surgeon, 103 Military Hospital, Vietnam Military Medical University, Hanoi 12108, Vietnam; surgeonvuanhdung@gmail.com; 11 Department of Functional Diagnosis, 103 Military Hospital, Vietnam Military Medical University, Hanoi 12108, Vietnam; phamngocthaovmmu@gmail.com

**Keywords:** dioxin, histopathological change, liver enzyme, liver damage, Vietnam

## Abstract

We investigated changes in some laboratory indices and the liver histology of chronic hepatitis patients who were exposed to dioxin. In 2014, we collected liver biopsy samples for histopathological examination from 33 chronic hepatitis patients living around the Da Nang Airbase, which is a dioxin-contaminated area due to the herbicide spraying in Vietnam. Dioxin exposure was measured by its levels in the blood. METAVIR classification was used to clarify the liver fibrosis stage. Laboratory tests included ten biochemical and six hematological indices that were measured in the blood. A regression linear model and binary logistic regression were used for data analysis. The observed alterations in the liver at the histological level mainly comprised hydropic degenerative hepatocytes, lymphocytes and polynuclear leukocytes surrounding the liver cells and granular and lipoic degeneration. In addition, increased TCDD levels were associated with increasing aminotransferase (AST), alanine aminotransferase, protein and total bilirubin levels and liver fibrosis stage. Similarly, increased TEQ-PCDD/Fs levels were associated with higher levels of AST and protein and liver fibrosis stage. In conclusion, dioxin exposure altered the liver histology and increased some biochemical marker indices and the liver fibrosis stage of chronic hepatitis patients living in dioxin-contaminated areas in Da Nang, Vietnam.

## 1. Introduction

During the Vietnam War from 1961 to 1972, the US Army carried out an herbicide spraying campaign with a huge amount of Agent Orange, which contained 2,3,7,8-tetrachlorodibenzo-p-dioxin (TCDD), in the southern areas of Vietnam. The impacts of the dioxin that originated from Agent Orange have been reported in both environmental and human health, even though the campaign ended over 50 years ago. Dioxin is absorbed into the body in various ways, including via the mucosal, skin, respiratory and gastrointestinal tracts; then, the circulatory system helps to distribute the dioxin around the other organs in the body [1]. Dioxin is insoluble in water and once it is absorbed into the bloodstream, it only exists in the blood for a short time before accumulating in fatty tissues and the liver. Moreover, the liver plays an essential role in metabolism and in immobilizing and inactivating the internal and external toxins within the human body. Therefore, it has been suggested that the liver is more vulnerable to damage that is caused by toxification from environmental toxins, including Agent Orange.

In previous studies, it has been reported that the liver is the main target of the toxic effects that are caused by TCDD exposure [2,3,4,5,6]. Serdar et al., (2014) investigated the effects of polychlorinated biphenyls (PCBs) and organochlorine pesticides (OCGs), which have similar toxicity to dioxin, on blood biochemistry markers. They showed that increased exposure to PCBs and OCGs is associated with increased levels of aspartate aminotransferase (AST), alanine aminotransferase (ALT) and gamma-glutamyl transpeptidase (GGT) [7]. Increased liver enzyme levels, including transaminases and GGT, have also been reported among residents who were exposed to TCDD by the Seveso accident [2]. An increased prevalence of liver disease, including liver cirrhosis, has been associated with exposure to dioxin originating from Agent Orange in Vietnam [3,4,5,6]. In addition, microscopic examinations (histological examinations) have also reported that dioxin causes morphological lesions in hepatic parenchymal cells, such as parenchymal degeneration [8], and electron microscopical evaluations in experimental studies have reported nucleus, mitochondria, rough endoplasmic reticulum, cytostomes and bile canaliculus in the cytoplasm [9]. However, limited studies have investigated the effects of dioxin exposure on the human liver using microscopes or electron microscopes, except a study of patients exposed to PCBs and dioxin by the soot from the Binghamton State Office Building incident. They found that TCDD and PCBs induce morphologic alterations in liver cells, as indicated by changes in the hepatic parenchymal cell cytoplasm, endoplasmic reticulum or mitochondria of patients who showed mild liver enzyme levels, including GGT, AST and ALT. These alterations were similar to those seen in animals that were exposed to PCBs and dioxin via feeding, although they did not produce the same microscopical findings and the number of subjects in the study was small, with only three patients [10]. From these results, we hypothesized that alterations in liver morphology at the histological level among chronic hepatitis patients were caused by dioxin exposure from Agent Orange.

Therefore, in the present study, we investigated the associations between dioxin exposure and some biomarker indices, including liver enzyme levels, lipid metabolism, hematology and histological alterations in the livers of patients who were diagnosed with chronic hepatitis and exposed to dioxin that originated from Agent Orange in Vietnam.

## 2. Materials and Methods

### 2.1. Study Areas and Subjects

The Da Nang Airbase is located inside the city of Da Nang in a condemned neighborhood that was used to store herbicide in Vietnam and is considered to be a hot spot for dioxin contamination [11]. Hatfield Consultants monitor environmental hazards, including heavy metals and organochlorines, and reported that the TCDD concentrations in soil samples collected inside the airbase from December 2006 to January 2009 ranged from 858 to 361,000 pg/g dry weight. In addition, elevated dioxin concentrations have been found not only in the blood of individuals working on the airbase but also in that of residents living around the airbase [12,13].

A total of 40 patients living in the hot spot of dioxin contamination within Da Nang city were selected from 17 military hospitals from August 2014 to January 2015. The criteria for the subject recruitment were as follows: (i) patients volunteered to participate the survey; (ii) patients were in the age range of 18–70 years; (iii) the liver enzyme levels of the patients were continuously high or were high for intermittent periods of at least six months (indicated by live biopsies to diagnose chronic hepatitis) [14]; (iv) patients lived in the hot spot of dioxin contamination (Da Nang Airbase) for at least five years and the toxic equivalent (TEQ) of polychlorinated dibenzo-p-dioxins (PCDDs) and polychlorinated dibenzofurans (PCDFs) TEQ levels in their serum was higher than the background levels (>9.4 pg-TEQ/g lipid) [15]; (vi) patients were not diagnosed with chronic hepatitis due to other factors, including the hepatitis B and C viruses, alcohol, drugs and autoimmune diseases, and were contraindicated for liver biopsy [14]. The patients were diagnosed with chronic hepatitis based on histopathological findings: mono leukocytes (mainly lymphocytes) appearing in portal spaces and fibrosis imaging [16,17]. Of these, seven patients refused to participate in the liver biopsy procedure. Therefore, the final number of subjects in our data analysis was 33 patients.

Information about the patients was collected, including their age, gender, smoking status, alcohol consumption (alcohol drinking history) and the number of years that they had lived in dioxin contaminated areas until the recruitment time.

Written informed consent was obtained from all of the participants, according to a process that had been reviewed and approved by the Ethics Council of the Vietnam Military Medical University (Number: 33.13/11-15).

### 2.2. Dioxin Measurements

A 40 mL sample of whole blood was collected from each patient. Then, the samples were centrifuged to separate the serum and stored at −30 °C in the Vietnam Russia Tropical Center until analysis. Dioxin measurement was performed in the dioxin laboratory at the Chemical–Environment Sub-Institute of the Vietnam Russia Tropical Center. The laboratory has been recognized for compliance with ISO/IEC 17025: 2017 and has received VILAS 856. Firstly, the serum samples were added to the internal standards of the 13C12-PCDD/PCDF isotope and then, under a procedure of liquid–liquid extraction, to the hexane–ethanol mixture. The extraction was recovered by n-hexane solution. The PCDD/PCDF fractions were separated on a dedicated activated carbon column, which was followed by cleaning steps on a “multi-layer column” containing silica gel, acid-impregnated silica gel and alkaline silica gel. Finally, the PCDD/PCDF fractions were separated through an aluminum oxide column after adding a standard of the 13C12-PCDD isotope to determine recovery efficiency. Dioxin measurement was followed by high-resolution gas chromatography/high-resolution mass spectrometry (GC-MS) using gas chromatography (Agilent 7890A) and high-resolution mass spectrometry (AutoSpec Premier P834). Certified reference materials from the National Institute of Standards and Technology (NIST) were regularly analyzed for quality control. The detection limits for each congener (in picograms per gram of wet weight) in the serum were as follows: TCDD, 0.013; 1,2,3,7,8-pentachlorodibenzo-p-dioxin (1,2,3,7,8-PCDD), 0.013; 1,2,3,4,7,8-hexachlorodibenzo-p-dioxin (1,2,3,4,7,8-HCDD), 0.033; 1,2,3,6,7,8-hexachlorodibenzo-p-dioxin (1,2,3,6,7,8-HCDD), 0.033; 1,2,3,7,8,9-hexachlorodibenzo-p-dioxin (1,2,3,7,8,9-HCDD), 0.033; 1,2,3,4,6,7,8-heptachlorodibenzo-p-dioxin (1,2,3,4,6,7,8-HpCDD), 0.033; octachlorodibenzo-p-dioxin (OCDD), 0.033; 2,3,7,8-tetrachlorodibenzofuran (2,3,7,8-TCDF), 0.013; 1,2,3,7,8-pentachlorodibenzofuran (1,2,3,7,8-PCDF), 0.013; 2,3,4,7,8-pentachlorodibenzofuran (2,3,4,7,8-PCDF), 0.033; 1,2,3,4,7,8-hexachlorodibenzofuran (1,2,3,4,7,8-HCDF), 0.033; 1,2,3,6,7,8-hexachlorodibenzofuran (1,2,3,6,7,8-HCDF), 0.033; 1,2,3,7,8,9-hexachlorodibenzofuran (1,2,3,7,8,9-HCDF), 0.033; 2,3,4,6,7,8-hexachlorodibenzofuran (2,3,4,6,7,8-HCDF), 0.033; 1,2,3,4,6,7,8-heptachlorodibenzofuran (1,2,3,4,6,7,8-HCDF), 0.033; 1,2,3,4,7,8,9-heptachlorodibenzofuran (1,2,3,4,7,8,9-HpCDF), 0.033; octachlorodibenzofuran (OCDF), 0.033. The limit of quantitation for each dioxin congener (pg/g) in the serum was as follows: TCDD, 0.017; 1,2,3,7,8-PeCDD, 0.0833; 1,2,3,4,7,8-HxCDD, 0.0833; 1,2,3,6,7,8-HxCDD, 0.0833; 1,2,3,7,8,9-HxCDD, 0.0833; 1,2,3,4,6,7,8-HpCDD, 0.0833; OCDD, 0.1667; 2,3,7,8-TCDF, 0.017; 1,2,3,7,8-PeCDF, 0.0833; 2,3,4,7,8-PeCDF, 0.0833; 1,2,3,4,7,8-HxCDF, 0.0833; 1,2,3,6,7,8-HxCDF, 0.0833; 2,3,4,6,7,8-HxCDF, 0.0833; 1,2,3,7,8,9-HxCDF, 0.0833; 1,2,3,4,6,7,8-HpCDF, 0.0833; 1,2,3,4,7,8,9-HpCDF,: 0.0833; OCDF, 0.1667. The TEQs of the seven PCDD and ten PCDF congeners were calculated as the sum of all values, which were obtained by multiplying each congener concentration by its toxic equivalent factor from the WHO 2005-TEF [18].

### 2.3. Laboratory Tests

Ten biochemical and six hematological parameters were measured in the serum of each patient selected from 17 military hospitals in Da Nang, Vietnam. An amount of 2 mL of venous blood was withdrawn from each patient for biochemical testing. Liver enzymes, consisting of GGT, AST and ALT, and other biochemical markers, including protein, cholesterol, triglyceride, total bilirubin, glucose, urea and creatinine, in the serum were measured using the fully automated Hitachi 912 Chemistry Analyzer (Roche Diagnostics, Mannheim, Germany). All reagent kits were obtained from Roche Diagnostics, India. Similarly, 2 mL of venous blood was used to measure the six hematological indices, including red blood cell count, hemoglobin, leukocytes, neutrocytes, lymphocytes and platelets, using the DxH 600 Hematology Analyzer (Beckman coulter, Seattle, WC, USA).

### 2.4. Histopathological Examination

Liver histopathology plays a critical role in diagnosing and finding the causes of liver disease, as well as being the gold standard for diagnosing and assessing liver damage. We used 16-gauge biopsy needles (DeltaCut, Pajunk Medizintechnologie GmbH, Baden-Württemberg, Germany) with the needle length of the gun adjusted from 15 to 22 mm to collect the liver biopsy samples. The samples were selected for histopathology examination when they met the following criteria: the specimen size was >1.5 cm with six portal spaces and a diameter of 1.4 mm. Then, the specimens were fixed with 10% formol, cast with paraffin and cut into 4-µm slices to form the template before being stained with hematoxylin–eosin. The Olympus BX51 electron microscope (Olympus, Tokyo, Japan) was used to examine the biopsy samples.

**Figure 1:**
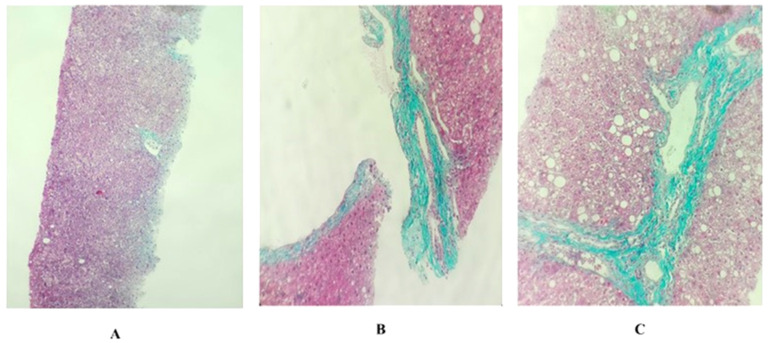
Histopathology of liver tissues under 10× microscope. The METAVIR fibrosis staging system (F0, F1, F2): (**A**) F0 (the core needle biopsy shows liver tissue with two central veins, including the infiltration of some chronic inflammatory cells, many hepatocytes with hydropic degeneration and a few hepatocytes with fatty degeneration, but there is no appearance of portal zones); (**B**) F1 (the core needle biopsy shows liver tissue with one portal zone and no central veins, including the infiltration of numerous chronic inflammatory cells, a strong development of connective tissue surround portal zone, the bile duct is expanded and many hepatocytes contain fatty degeneration, but there is no appearance of bridging); (**C**) F2 (the core needle biopsy shows liver tissue with one or two portal zones and probably one central vein, including the infiltration of numerous chronic inflammatory cells, a strong development of connective tissue surrounding portal zones, many bile ducts are expanded, many hepatocytes contain large fatty degeneration and there is probably the appearance of bridging between central vein and portal zone).

The METAVIR scoring system is simple and the most commonly used system in clinical practice. According to METAVIR, there are five stages of fibrosis, as follows: F0, no fibrosis; F1, portal fibrosis without spaces; F2, portal fibrosis and several bridges; F3, fibrosis with multiple bridges or bridge fibrosis, F4, cirrhosis [19]. Figure 1 displays the characteristics of the F0, F1 and F2 stages of fibrosis.

### 2.5. Statistical Analysis

SPSS v. 21.0 for Windows (IBM Corp., Armonk, NY, USA) was used for the data analysis. The concentrations of TCDD and TEQ-PCDD/Fs and the laboratory indices were logarithmically transformed (base 10) to improve normality. The relationships between the TCDD and TEQ-PCDD/Fs levels and the laboratory indices were analyzed using a regression linear model after adjusting for covariates, including gender, age and smoking status. At this time, variables were selected as covariates if they correlated with at least one biochemical or hematological marker, based on a Pearson’s correlation analysis, or if the groups differed significantly in at least one biochemical or hematological marker (Student’s *t*-test, *p* < 0.05). Since the number of the patients who showed the F0 METAVIR grade was small in the present study, we divided the subjects into positive and negative METAVIR scores as follows: positive METAVIR score for those who showed liver fibrosis at stage F2 and negative METAVIR scores for those who showed liver fibrosis at stages F0 or F1. Then, the associations between dioxin exposure, as indicated by the TCDD and TEQ-PCDD/Fs levels, and positive METAVIR scores were analyzed using a binary logistic regression model after adjusting for the same confounding factors as above. A *p*-value of < 0.05 was considered statistically significant.

## 3. Results

### 3.1. Characteristics of the Subjects

The characteristics of the study subjects are shown in Table 1. The average age of the subjects was 46.3 years, with the rate of male subjects and patients who were smokers being 48.5% of total subjects. None of the subjects consumed alcoholic beverages. The mean length of time that patients lived in a hot spot for dioxin contamination was 26.0 years. The mean body mass index of the subjects was within the normal range (<25). For the METAVIR score, the percentage of patients with the F0 grade was the lowest, which accounted for 17.3% of the subjects. The percentage of patients with F1 and F2 grades were 42.4% and 30.3%, respectively. The geometrical mean of the TCDD and TEQ-PCDD/Fs levels in the blood was 15.7 pg/g lipid and 42.5 pg-TEQ/g lipid, which was found to be seven or eight time higher than those in unsprayed areas of Vietnam [20].

### 3.2. Associations between TCDD and TEQ-PCDD/Fs Exposure and Laboratory Indices

The associations between dioxin exposure, as indicated by the levels of TCDD and TEQ-PCDD/Fs in the blood, and the biochemical and hematological indices were analyzed using a regression linear model after adjusting for gender, age and smoking status. The results are illustrated in Table 2.

The TCDD exposure levels had significant and positive associations with AST, ALT, total bilirubin and protein levels (*p* < 0.05). Similarly, the TCDD exposure levels had borderline significant and positive associations with GGT levels. However, no significant associations between the TCDD exposure levels and the other biomarker indices were found (Table 2).

Similarly, the TEQ-PCDD/Fs levels were significantly and positively associated with AST and protein levels (*p* < 0.05). Increased TEQ-PCDDs/Fs exposure levels had borderline significant associations with increased ALT levels (*p* = 0.052). No significant associations were found between the TEQ-PCDD/Fs exposure levels and GGT or the other biomarker indices measured in the present study (Table 2).

### 3.3. The Characteristics of Histopathological Damage

Chronic liver injury at the histological level was detected in almost all biopsy samples. The results are displayed in Table 3. The alterations of hydropic degenerative hepatocytes and lymphocytes and polynuclear leukocytes surrounding the liver cells were observed in all samples. Granular and lipoic degeneration was found in 32 of the total 33 samples, which accounted for 97% of the subjects. There were four cases (12.1%) that showed changes in hepatocytes eosinophils. Other indications of chronic liver disease, such as Mallory bodies, pigmentation, lipo-granuloma, megamitochondria and venous obstruction, were not observed in the present study (Table 3).

### 3.4. Associations between TCDD and TEQ-PCDD/Fs Exposure and METAVIR Scores

METAVIR scores were used to clarify the degree of liver fibrosis. The highest fibrosis level in the present study was F2, which accounted for 30.3% of the subjects. The percentage of F1 and F2 grades were 42.4% and 27.3%, respectively. No F3 and F4 grades were found in the samples from this group, suggesting that there were no samples that showed a fibrous expansion of portal areas with marked bridging, marked bridging with occasional nodules (incomplete cirrhosis) or cirrhosis [19] (Table 1). To find the effects of dioxin exposure on liver fibrosis level, the relationships between TCDD and TEQ-PCDD/Fs exposure levels and positive METAVIR scores were analyzed using a binary logistic regression model, which was adjusted for age, gender and smoking status. The results are shown in Table 4. The results showed that increased exposure levels of TCDD and TEQ-PCDD/Fs were significantly associated with increased odd ratios of positive fibrosis grades (TCDD, OR = 5.8 and *p* = 0.007; TEQ-PCDD/Fs, OR = 3.8 and *p* = 0.021) (Table 4).

## 4. Discussion

In this study, we found that increased exposure to TCDD was significantly associated with increased serum AST and ALT levels. TCDD exposure levels also had borderline significant and positive associations with GGT levels. Similarly, increased exposure to TEQ-PCDD/Fs had significant or borderline significant associations with increased serum AST and ALT levels. These results suggest that dioxin exposure, as indicated by levels of TCDD and TEQ-PCDD/Fs in the blood, increases serum liver enzyme levels, particularly AST and ALT.

In previous studies, investigations were conducted on the effects of PCBs and OCGs, which have similar toxicity to dioxin, on blood biochemistry markers during the National Health and Nutrition Examination Survey (NHANES 2003–2004) in the US. Serdar et al., (2014) reported that increased exposure to PCBs and OCGs is associated with increased AST, ALT and GGT levels [7]. Increased liver enzyme levels, including transaminases and GGT, were also reported among residents who were exposed to TCDD during the Seveso accident [2]. In a study in Germany, Triebig et al., (1998) recruited 76 former workers from a non-ferrous metal recycling facility who showed elevated levels of TEQ-PCDD/Fs (median = 42 ppt, range = 13–281). The authors reported significant and positive associations between dioxin exposure and liver enzyme levels, indicated by alanine aminotransferase, albeit no such associations were observed with serum cholesterol levels or high-density lipoprotein cholesterol (HDL) in this study [21]. In contrast, these associations were not found in other studies [22,23]. In a group of 138 former chemical workers who had been potentially exposed to TCDD following the 1953 trichlorophenol autoclave accident, there were no significant associations between TCDD exposure and liver enzyme (AST, ALT and GGT), serum glucose, lipid metabolism (cholesterol, triglyceride, HDL and low-density lipoprotein cholesterol (LDL)), hematology or coagulation parameters, except platelets [23]. In this study, we also found that there were no associations between TCDD and TEQ-PCDD/Fs exposure and hematological parameters (glucose, urea and creatinine) or lipid parameters (serum cholesterol and triglyceride levels); however, HDL and LDL levels were not measured in the present study. Positive associations were found between exposure to TCDD and TEQ-PCDD/Fs and plasma protein and total bilirubin levels. In addition, alterations in liver enzyme, lipid metabolism, total protein, bilirubin, blood cell, hemoglobin and plasma protein levels were induced by TCDD exposure, which was most probably associated with food consumption and dietary intake [24]. Therefore, statistical associations between TCDD and TEQ-PCDD/Fs exposure that originated from Agent Orange and liver enzyme parameters (glucose and blood cells), lipid parameters (cholesterol and triglyceride) and other biomarker indices in serum should be re-evaluated in a follow-up study after controlling food intake.

In addition, in the present study, we found alterations in liver morphology at the histological level, as indicated by granular degeneration, hydropic degeneration, lipoic degeneration and lymphocytes and polynuclear leukocytes surrounding the liver cells, in most biopsy samples from chronic hepatitis patients who were exposed to high levels of dioxin. Our report was similar to the results from the previous experimental studies. After investigating the effects of TCDD on the liver at the histological level, the previous studies reported that parenchymal degeneration and the vacuolization of hepatocytes was observed in the rats that received TCDD [8] and degenerated hepatocytes, lobular inflammation and marked fat accumulation in TCDD-treated mice [25], although different doses of TCDD was used between the two studies. These results suggest that dioxin exposure causes alterations in liver morphology at the histological level, which was clearly exhibited by inflammation and degenerated hepatocytes.

Other indications of chronic liver disease, such as Mallory bodies, pigmentation, granulomatosis, cytoplasmic alterations, megamitochondria and venous obstruction, were also assessed but were not seen in any subjects in the present study. In a previous study, it was reported that alterations in cytoplasm, fatty change, bile duct hyperplasia and pigmentation were found in the livers of rats that were exposed to TCDD [26]. In a critical review of the histopathological findings of 118 papers on endocrine and non-endocrine hepatic toxicity in fish, Wolf et al., (2018) also reported that exposure to endocrine and non-endocrine hepatic toxicity, such as TCDD, induced cytoplasmic alterations, including Mallory bodies, pigmentation, granulomatosis, megamitochondria and venous obstruction [27]. However, these alterations were not detected in the present study. This could partly be explained by the expression of the effects of dioxin exposure on morphology at the histological level based the exposure levels, the length of exposure to dioxin and the location from which the tissues were collected [28]. In addition, the number of subjects in this study was small, which could also have affected results. Therefore, further studies with higher numbers of subjects are required to investigate the effects of dioxin exposure on histological changes in the liver.

Furthermore, in the present study, we found that increased exposure to TCDD and TEQ-PCDD/Fs was associated with increased METAVIR scores. We found that the most common score was F1, followed by F0 and F2. No cases of F3 and F4 scores were detected. The Vietnam Ministry of Health has classified primary liver cancer as on the list of diseases, malformations and congenital abnormalities that are associated with exposure to toxic chemicals/dioxin [29]. An increased prevalence of mortality from all causes of death, including liver cancer and liver cirrhosis, associated with exposure to dioxin that originated from the use of Agent Orange in Vietnam has also been reported [3,4,5,6]. Therefore, dioxin that originated from Agent Orange could potentially cause very serious liver damage, but no such serious liver damage was observed in the current study. It is possible that the above studies were mainly conducted on veterans, most of whom were directly exposed to the dioxin that was sprayed by the US during the war, and that these studies were conducted a relatively long time ago. Moreover, our study was conducted on people living in the dioxin contamination hot spot in Da Nang, who had longer periods of exposure to dioxin but at a lower concentration that decreased over time (due to the natural decomposition of dioxin and the dioxin detoxification process). Furthermore, the determination of the degree of liver fibrosis, i.e., significant or severe fibrosis, is suggested to be very important to establish the correct treatment method and to screen for complications. Most patients with significant fibrosis (≥F2) need immediate treatment to avoid progression to severe fibrosis. For severe fibrosis (≥F3), patients need special monitoring and screening for complications (liver cancer, gastrointestinal bleeding due to esophageal varices rupture, etc.). Therefore, regular health checks are necessary for people living in hot spots of dioxin contamination in Vietnam to establish the correct therapy to treat patients with F2 grade fibrosis and to prevent the progression of patients with F0 and F1 grade fibrosis.

The present study was the first study to report the effects of dioxin exposure that originated from Agent Orange on human health at the histological level in Vietnam. It is generally accepted that the toxicity of TCDD is induced mainly via the activation of the Ah receptor binding to the xenobiotic response element of target genes [30], leading to the induction or suppression of the transcription of numerous genes that have been linked to cancer development due to changes in tumor suppressor proteins, oncogenes, growth factors and cell cycle proteins, among other factors [31]. It has been suggested that TCDD promotes tumor progression in vivo by directly targeting mitochondrial transcription and the induction of mitochondrial stress signaling [32]. In addition, mitochondrial dysfunctions are frequently described as early and initiating events in various chronic pathological conditions in different tissues and organs, including the liver, brain and heart, and have been suggested as biomarkers for the early detection of cancer [33]. Alterations in the liver at the ultrastructural level, particularly in mitochondria, have been found in chronic hepatitis patients who were exposed to dioxin [10]. Therefore, we plan to investigate the associations between dioxin exposure and alterations in the liver at the ultrastructural level in the present subjects. Health regular checks, including checks for liver cancers and chronic diseases in other organs, is required in all subjects.

*Limitations*: In this study, we investigated alterations at the histological level in the livers of patients who were diagnosed with chronic hepatitis and were exposed to high levels of dioxin. However, our study had some limitations. We did not have a control group to compare the effects of dioxin exposure on the liver at the histological level. We initially selected 33 chronic hepatitis B patients living in unsprayed areas of Vietnam as the control group. However, there were only three male patients and no patients with F0 grade fibrosis and 22 patients (66.7%) showed higher than F1 grade fibrosis according to the METAVIR scale. Therefore, we decided that this group was not suitable as a control group for the present study. Other factors, such as food intake, were also not controlled in the present study, which could have affected the results of the laboratory tests. Another limitation of the present study was its relatively small sample size, particularly for the comparison of TCDD and TEQ-PCDD/Fs levels among different liver fibrosis grades.

## 5. Conclusions

This study showed alterations at the histological level in the livers of patients with chronic hepatitis who were exposed to dioxin that originated from Agent Orange. These alterations mainly comprised hydropic degenerative hepatocytes, lymphocytes and polynuclear leukocytes surrounding the liver cells and granular and lipoic degeneration. Furthermore, increased TCDD levels were associated with increased AST, ALT, protein and total bilirubin levels and liver fibrosis stage. Similarly, increased TEQ-PCDD/Fs levels were associated with higher levels of AST, protein and liver fibrosis stage. Regular health checks, including checks for dioxin-related diseases, should be required for all subjects living in dioxin contamination areas in Vietnam.

## Figures and Tables

**Table 1:** Characteristics of the study subjects.

	Characteristic	Unit	Mean (SD), N [%]
Subjects			
	Age	year	46.3 (12.1)
	Ratio of Male	% male	16 [48.5%]
	Smoking Status	% smoker	16 [48.5%]
	Alcohol Consumption	% drinking alcohol	0 [0.0%]
	Length stayed in dioxin contaminated areas	Year	26.0 (10.6)
	BMI		21.7 (2.1)
METAVIR Score			
	F0	%	9 [17.3%]
	F1	%	14 [42.4%]
	F2	%	10 [30.3%]
Dioxin Concentration in Blood			
	TCDD	pg/g lipid	15.7 (3.7) *
	TEQ-PCDD/Fs	pg-TEQ/g lipid	42.5 (2.5) *

N, number of subjects; SD, standard deviation; BMI, body mass index; TCDD, 2,3,7,8-tetrachlorodibenzo-p-dioxin; PCDD/Fs, polychlorinated dibenzo-p-dioxins and polychlorinated dibenzo furans; TEQ, toxic equivalent quantity; *, geometrical mean and geometrical standard.

**Table 2:** Associations between TCDD and TEQ-PCDD/Fs concentration and biomarker indices.

		TCDD			TEQ-PCDD/FS		
Markers	N	β	95% CI (Lower, Upper)	*p*	β	95% CI (Lower, Upper)	*p*
Aspartate Aminotransferase (AST)	33	0.414	(0.071, 0.758)	0.020	0.393	(0.046, 0.741)	0.028
Alanine Aminotransferase (ALT)	33	0.359	(0.009, 0.709)	0.045	0.349	(−0.003, 0.702)	0.052
Gamma-Glutamyl Transpeptidase (GGT)	33	0.340	(−0.020, 0.699)	0.063	0.298	(−0.068, 0.664)	0.107
Total Bilirubin	33	0.389	(0.030, 0.748)	0.035	0.283	(−0.091, 0.656)	0.133
Glucose	33	−0.05	(−0.417, 0.318)	0.79	−0.14	(−0.502, 0.229)	0.45
Urea	33	0.031	(−0.318, 0.381)	0.855	−0.013	(−0.364, 0.337)	0.938
Creatinin	33	−0.011	(−0.284, 0.262)	0.933	0.062	(−0.211, 0.334)	0.648
Protein	33	0.421	(0.104, 0.738)	0.011	0.351	(0.021, 0.680)	0.038
Cholesterone	33	0.029	(−0.334, 0.392)	0.871	0.006	(−0.358, 0.370)	0.974
Triglyceride	33	0.043	(−0.334, 0.420)	0.816	0.104	(−0.272, 0.480)	0.576
Red Blood Cells	33	0.137	(−0.200, 0.474)	0.412	0.078	(−0.263, 0.418)	0.644
Hemoglobin	33	0.003	(−0.288, 0.294)	0.985	−0.024	(−0.316, 0.267)	0.866
Leukocytes	33	−0.031	(−0.420, 0.357)	0.870	−0.225	(−0.605, 0.155)	0.236
Neutrocytes	33	−0.201	(−0.582, 0.181)	0.291	−0.240	(−0.619, 0.139)	0.205
Lymphocytes	33	0.234	(−0.146, 0.614)	0.218	0.299	(−0.075, 0.673)	0.113
Platelets	33	−0.172	(−0.549, 0.206)	0.360	−0.127	(−0.508, 0.254)	0.500

TCDD, 2,3,7,8-tetrachlorodibenzo-p-dioxin; PCDD/Fs, polychlorinated dibenzo-p-dioxins and polychlorinated dibenzo furans; TEQ, toxic equivalent quantity; N, number of subjects; β, standardized beta; 95% CI, 95% confidence interval. Confounding factors: gender; age; smoking status.

**Table 3:** Histopathological damage among chronic hepatitis patients who were exposed to dioxin.

Histopathological Damage	Yes N (%)	No N (%)
Granular Degeneration	32 (97.0)	1 (3.0)
Hydropic Degeneration	33 (100)	0 (0.0)
Lipoic Degeneration	32 (97.0)	1 (3.0)
Lipogranuloma	0 (0.0)	33 (100)
Lymphocytes and Polynuclear Leukocytes Surrounding the Liver Cells	33 (100)	0 (0.0)
Mallory Bodies	0 (0.0)	33 (100)
Pigmentation	0 (0.0)	33 (100)
Megamitochondria	0 (0.0)	33 (100)
Changes in Acidophil Hepatocyte	4 (12.1)	29 (87.9)
Venous Obstruction	0 (0.0)	33 (100)

N, number of subjects.

**Table 4:** Relationships between TCDD and TEQ-PCDD/Fs exposure and positive METAVIR scores (F2).

	OR	SE	95% CI (Lower, Upper)	*p*
TCDD	5.9	0.7	(1.6, 21.4)	0.007
TEQ-PCDDs/Fs	3.9	0.6	(1.2, 12.1)	0.021

TCDD, 2,3,7,8-tetrachlorodibenzo-p-dioxin; TEQ, toxic equivalent quantity; PCDD/Fs, polychlorinated dibenzo-p-dioxins and polychlorinated dibenzo furans; OR, odd ratio; SE, standard error; 95% CI, 95% confidence interval. Covariates: age; gender; smoking status.

## Data Availability

The data presented in this study are available on request to the corresponding author. The data are not publicly available due to the personal information (laboratory indices and the liver histology of the subjects).

## References

[1] Schecter A., Dai L.C., Thuy L.T., Quynh H.T., Minh D.Q., Cau H.D., Phiet P.H., Nguyen N.T., Constable J.D., Baughman R. (1995). Agent Orange and the Vietnamese: The persistence of elevated dioxin levels in human tissues. Am. J. Public Health.

[2] Pocchiari F., Silano V., Zampieri A. (1979). Human health effects from accidental release of tetrachlorodibenzo-p-dioxin (TCDD) at Seveso, Italy. Ann. N. Y. Acad Sci..

[3] Tamburro C.H. (1992). Chronic liver injury in phenoxy herbicide-exposed Vietnam veterans. Environ. Res..

[4] O’Toole B.I., Marshall R.P., Grayson D.A., Schureck R.J., Dobson M., Ffrench M., Pulvertaft B., Meldrum L., Bolton J., Vennard J. (1996). The Australian Vietnam Veterans Health Study: II. self-reported health of veterans compared with the Australian population. Int. J. Epidemiol..

[5] Kang H.K., Dalager N.A., Needham L.L., Patterson D.G., Lees P.S., Yates K., Matanoski G.M. (2006). Health status of Army Chemical Corps Vietnam veterans who sprayed defoliant in Vietnam. Am. J. Ind. Med..

[6] Yi S.W., Ryu S.Y., Ohrr H., Hong J.S. (2014). Agent Orange exposure and risk of death in Korean Vietnam veterans: Korean Veterans Health Study. Int. J. Epidemiol..

[7] Serdar B., LeBlanc W.G., Norris J.M., Dickinson L.M. (2014). Potential effects of polychlorinated biphenyls (PCBs) and selected organochlorine pesticides (OCPs) on immune cells and blood biochemistry measures: A cross-sectional assessment of the NHANES 2003-2004 data. Environ. Health.

[8] Czepiel J., Biesiada G., Gajda M., Szczepański W., Szypuła K., Dabrowski Z., Mach T. (2010). The effect of TCDD dioxin on the rat liver in biochemical and histological assessment. Folia Biol..

[9] Fowler B.A., Lucier G.W., Brown H.W., McDaniel O.S. (1973). Ultrastructural changes in rat liver cells following a single oral dose of TCDD. Environ. Health Perspect..

[10] Schecter A., Tiernan T., Schaffner F., Taylor M., Gitlitz G., VanNess G.F., Garrett J.H., Wagel D.J. (1985). Patient fat biopsies for chemical analysis and liver biopsies for ultrastructural characterization after exposure to polychlorinated dioxins, furans and PCBs. Environ. Health Perspect..

[11] Dwernychuk L.W. (2005). Dioxin hot spots in Vietnam. Chemosphere.

[12] Hatfield Consultants, Office of National Committee 33, 2009 Comprehensive Assessment of Dioxin Contamination in Da Nang Airport, Viet Nam: Environmental Levels, Human Exposure and Options for Mitigating Impacts. (North Vancouver, BC, CANADA V7P0A3). http://www.hatfieldgroup.com/services/contaminant-monitoring-agent-orange/hatfield-agent-orange-reports-and-presentations/.

[13] Tai P.T., Nishijo M., Kido T., Nakagawa H., Maruzeni S., Naganuma R., Anh N.T., Morikawa Y., Luong H.V., Anh T.H. (2011). Dioxin concentrations in breast milk of Vietnamese nursing mothers: A survey four decades after the herbicide spraying. Environ. Sci. Technol..

[14] Ministry of Health, Vietnam (2014). Decision Number 5448/ 5448/QĐ-BYT on the Guidance for Diagnosis, Treatment of Hepatitis B Disease (in Vietnamese: Quyết định số 5448/QĐ-BYT: Về việc ban hành hướng dẫn chẩn đoán, điều trị bệnh viêm gan vi rút B).

[15] Olaf P. (1998). PCDD/PCDF: Human background data for Germany, a 10-year experience. Environ Health Perspect..

[16] Jules L.D. (2015). Chronic Hepatitis. Harrison’s Principles of Internal Medicine.

[17] Maria G., Alessandra M., Gavino F. (2011). Chronic viral hepatitis: The histology report. Dig. Liver Dis..

[18] Van den Berg M., Birnbaum L.S., Denison M., De Vito M., Farland W., Feeley M., Fiedler H., Hakansson H., Hanberg A., Haws L. (2006). The 2005 World Health Organization reevaluation of human and mammalian toxic equivalency factors for dioxins and dioxin-like compounds. Toxicol. Sci..

[19] Poynard T., Ratziu V., Benmanov Y., Di Martino V., Bedossa P., Opolon P. (2000). Fibrosis in Patients with Hepatitis C: Detection and Significance: Detection and Significance. Semin. Liver Dis..

[20] Van Luong H., Tai P.T., Nishijo M., Trung D.M., Thao P.N., Van Son P., Van Long N., Linh N.T., Nishijo H. (2018). Association of dioxin exposure and reproductive hormone levels in men living near the Bien Hoa airbase, Vietnam. Sci. Total Environ..

[21] Triebig G., Werle E., Päpke O., Heim G., Broding C., Ludwig H. (1998). Effects of dioxins and furans on liver enzymes, lipid parameters, and thyroid hormones in former thermal metal recycling workers. Environ. Health Perspect..

[22] Calvert G.M., Hornung R.W., Sweeney M.H., Fingerhut M.A., Halperin W.E. (1992). Hepatic and gastrointestinal effects in an occupational cohort exposed to 2,3,7,8-tetrachlorodibenzo-para-dioxin. JAMA.

[23] Ott M.G., Zober A., Germann C. (1994). Laboratory results for selected target organs in 138 individuals occupationally exposed to TCDD. Chemosphere.

[24] Greig J.B., Jones G., Butler W.H., Barnes J.M. (1973). Toxic effects of 2,3,7,8-tetrachlorodibenzo-p-dioxin. Food Cosmet. Toxicol..

[25] Lu H., Cui W., Klaassen C.D. (2011). Nrf2 protects against 2,3,7,8-tetrachlorodibenzo-p-dioxin (TCDD)-induced oxidative injury and steatohepatitis. Toxicol. Appl. Pharmacol..

[26] Maronpot R.R., Foley J.F., Takahashi K., Goldsworthy T., Clark G., Tritscher A., Portier C., Lucier G. (1993). Dose response for TCDD promotion of hepatocarcinogenesis in rats initiated with DEN: Histologic, biochemical, and cell proliferation endpoints. Environ. Health Perspect..

[27] Wolf J.C., Wheeler J.R. (2018). A critical review of histopathological findings associated with endocrine and non-endocrine hepatic toxicity in fish models. Aquat Toxicol..

[28] Dobrzyński M., Madej J.P., Leśków A., Tarnowska M., Majda J., Szopa M., Gamian A., Kuropka P. (2021). The Improvement of the Adaptation Process of Tocopherol and Acetylsalicylic Acid in Offspring of Mothers Exposed to TCDD. Animals.

[29] Ministry of Health, Vietnam (2008). The Decision Number 09/2008/QĐ-BYT: Promulgating the List of Diseases, Deformities, Deformities, RELATED TO EXPosure to Toxic Chemicals/Dioxins (in Vietnamsese Quyết định số 09/2008/QĐ-BYT: Ban hành Danh mục bệnh, tật, dị dạng, dị tật có liên quan đến phơi nhiễm với chất độc hóa học/dioxin).

[30] Fernandez-Salguero P.M., Hilbert D.M., Rudikoff S., Ward J.M., Gonzalez F.J. (1996). Arylhydrocarbon receptor-deficient mice are resistant to 2,3,7,8-tetrachlorodibenzo-pdioxin-induced toxicity. Toxicol. Appl. Pharmacol..

[31] Mead M.N. (2008). Cancer and TCDD: The mitochondrial connection. Environ. Health Perspect..

[32] Biswasm G., Srinivasan S., Anandatheerthavarada H.K., Avadhani N.G. (2008). Dioxin-mediated tumor progression through activation of mitochondria-to-nucleus stress signaling. Proc. Natl. Acad. Sci. USA.

[33] Modica-Napolitano J.S., Singh K.K. (2004). Mitochondrial dysfunction in cancer. Mitochondrion.

